# Androgens, aging, and prostate health

**DOI:** 10.1007/s11154-022-09730-z

**Published:** 2022-06-24

**Authors:** Karin Welén, Jan-Erik Damber

**Affiliations:** Department of Urology, Institute of Clinical Sciences, Sahlgrenska Center for Cancer Research, Sahlgrenska Academy, University of Gothenburg, Gothenburg, Sweden

**Keywords:** Benign prostate hyperplasia, Prostate cancer, Testosterone replacement therapy, Androgen signaling, Metabolic syndrome, Aging

## Abstract

Due to late onset hypogonadism (LOH), there is an increased usage of testosterone replacement therapy (TRT) in the aging male population. Since prostate is a target organ for androgens and anti-androgenic strategies are used to treat and palliate benign prostate hyperplasia (BPH) and prostate cancer (PC), the prevalence of both increases with age, the possible influence of TRT on prostate health becomes highly relevant. The present review summarizes existing data on the associations between endogenous hormone concentrations and prostate growth and concludes that circulating concentrations of androgens do not appear to be associated with the risks of development of BPH or initiation or progression of PC. The explanation for these findings relates to an apparent insensitivity of prostatic tissue to changes of testosterone concentrations within the physiological range.

## Introduction

In recent years there has been a significant increase of the use of testosterone treatment in men with “late onset hypogonadism” (LOH). From 2010 to 2013 in the US, approximately 80% of the testosterone prescriptions were for men between 40 and 74 years of age [1]. One reason for this is the awareness that LOH is associated with several significant health problems such as metabolic disorders, sexual dysfunction, osteopenia, and psychological stress [2]. Testosterone replacement therapy (TRT) has been used with the purpose to improve or even reverse these symptoms [3, 4]. TRT for LOH has however been questioned by the US food and drug administration (FDA) concerning the large increase in testosterone prescriptions, and how to define the group of patients with LOH in order to make more strict treatment recommendations. FDA also had concerns because of some support for an increased risk of cardiac problems, but not due to possible risks for prostate diseases [5], although conclusive evidence for its safety is still largely lacking.

The prostate gland is an androgen target organ. In clinical medicine, strategies to minimize the stimulatory influences of androgens are successfully used to treat common prostate disorders such as prostate cancer (PC) and benign prostate hyperplasia (BPH). Consequently, it has long been believed that higher levels of testosterone increase the risk of PC and BPH. Since TRT is increasingly used in the aged male population the influence of such therapy on prostate diseases is of major importance. The aim of the present review was therefore to elucidate and discuss the role of testosterone, aging, and TRT for prostate health.

## Benign prostate hyperplasia (BPH)

BPH is one of the most common diseases in the aging male. Its prevalence increases from 30 to 40% in the 4th decade of life to 70–80% in those older than 80 years [6–8]. BPH is a histological diagnosis caused by hyperproliferation of epithelial and stromal cells in the transition zone of the prostate [9]. The consequences of BPH are in most cases benign prostatic enlargement and lower urinary tract symptoms (LUTS). Observational studies from various parts of the world clearly demonstrate that older age is an important risk factor for development of BPH [7, 10]. The BPH initiating mechanisms are largely unknown. BPH is also considered as partly a hereditary disease. Male relatives and brothers to men below 64 years and undergoing surgery for BPH had a 4-6-fold increased age-specific risk for BPH surgery [11]. The hereditary form of BPH is associated with larger prostate volume and younger age of onset when compared with sporadic BPH [12].

### Androgens in BPH development

Androgens and androgen receptor (AR) signaling are necessary for normal prostate growth and homeostasis and have been linked to the development and maintenance of BPH. During puberty, testosterone is converted to dihydrotestosterone (DHT) by 5α reductase (5AR) within the prostate and that stimulates the prostate to grow to its adult size. In the normal prostate gland, this conversion of testosterone to DHT is of utmost importance for promoting the AR signaling which influences cell proliferation, differentiation, morphogenesis, and functional maintenance [13]. Support of a similar setting in BPH can be derived from the clinical experience that 5α reductase inhibitors (5ARI) are used to reduce DHT and thereby slow progression of clinical BPH [14]. In several observational studies, no associations with BPH and higher serum testosterone have been reported [15–17], suggesting that higher serum testosterone concentrations do not promote BPH. In one of these studies [15], high testosterone was associated with a decreased risk for BPH while low testosterone increased the risk. Also, estrogens have been suggested to play a role in regulating stromal-epithelial interactions involved in prostatic cellular growth [18], although the exact role remains unclear.

### Aging as a risk factor for BPH

Recently it has been suggested that several metabolic disorders more common in older ages, including hyperinsulinemia, dyslipidemia, and obesity might be important in the development of BPH [19–21]. Another key aspect on BPH development is inflammation [22], which is an important aspect of the metabolic syndrome (MetS) and associated with low testosterone and hyperestrogenism [23].

### The metabolic syndrome in BPH

MetS has been recognized as a cluster of some common medical disorders such as visceral obesity, glucose intolerance, hypertension, and dyslipidemia which increase the risk for cardiovascular disease and type 2 diabetes (T2DM) [24]. Previous studies suggest an association of MetS with BPH [25]. In an investigation in 158 men, individual MetS components such as T2DM, hypertension, obesity, high insulin, and low high density lipoprotein (HDL)-cholesterol levels were all found to be risk factors for the development of BPH [26]. In a more recent large cross sectional epidemiological study, it was shown that the risk of having MetS in men with clinical BPH was increased by 37% [27]. The mechanistic link between MetS and BPH is basically unknown. MetS is associated with low grade chronic inflammation with increased serum markers such as C-reactive protein (CRP) and proinflammatory cytokines [28, 29]. Inflammation in prostate biopsies has been associated with BPH progression [30] and increased CRP is associated with increased risk for BPH and LUTS [31]. The role of androgens in this context is unclear since MetS also is associated with low testosterone levels [32, 33].

### Senescence in the etiology of BPH

Aging tissue is associated with an increased number of senescent cells [34]. Senescence is a non-replicative state in which cells end up after irreparable DNA damage [35], and can be seen as a tumor suppressive mechanism. In a mouse model, reduced testosterone levels were associated with increased cell senescence and vascular remodeling, indicating that the low testosterone levels may indirectly influence the development of aging-related diseases [36]. Although senescent cells do not proliferate, they remain metabolically active and secrete inflammatory mediators [37], which contribute to the state of low-grade inflammation associated with age in both animals and humans [38]. Several studies have reported senescent prostate epithelial cells in almost all BPH samples investigated [39, 40], and the overlapping between the senescent secretome and mediators identified as promoting BPH suggests that this age-related feature may be an important factor in BPH pathology (as reviewed in [41]).

## Prostate Cancer

Worldwide, PC is the second most common malignancy in men and the fifth commonest cause of cancer mortality [42]. There are large variations in incidence and mortality rates according to geographical location with Sweden among the top rated regarding both incidence and mortality [43]. Based on the geographical variations of incidence and mortality, dietary factors have been thoroughly investigated, but the evidence so far remains inconclusive. The widespread usage of early detection strategies based on prostate specific antigen (PSA) measurements have resulted in increased incidence of PC in US and Europe with increase of indolent disease and a decrease of metastatic disease [44].

PC shows increased incidence with increasing age, and up to 80% of men over the age of 80 years harbor PC cells [45]. Besides age, well established risk factors for PC development are genetic predisposition, family history, and ethnicity [43]. An increased risk for high grade PC was observed in patients with the metabolic syndrome [46]. Obesity is associated with metabolic disturbances and is also associated with PC aggressiveness [47]. Chronic inflammation has been suggested as a potential link between metabolic disorders and PC risk [48].

### Androgens in prostate cancer development

Ever since the efficacy of castration, or androgen deprivation therapy (ADT), was proven 80 years ago, it has been the mainstay for the management of metastatic PC [49], indicating that testosterone is a driving factor for PC. It is known that both normal prostate epithelial cells and PC cells are dependent on androgens for their proliferation and survival [50, 51], even if androgen regulated epithelial proliferation in the normal prostate largely is mediated by stromal cells [52, 53]. However, despite comprehensive investigations, there are today no conclusive support for an increased risk of PC associated with higher testosterone in the physiological range. In 2016, a meta-analysis combining data from 20 separate studies, comprising samples from almost 20,000 men with a mean follow-up of 10 years, concluded that the levels of endogenous steroids were not associated with PC risk [54]. Contradicting this epidemiologic evidence, the Prostate Cancer Prevention Trial (PCPT) randomizing healthy men to a 5ARI (finasteride), blocking conversion of testosterone into DHT, reported that finasteride was associated with a reduction in diagnosed PC cases [55]. However, after 18-yr follow-up, no differences were observed between the two groups in overall and cancer specific mortality rates [56]. In principle, comparable results were obtained in the Reduction by Dutasteride of Prostate Cancer Events (REDUCE) phase III trial using another 5ARI, dutasteride, where men with a negative prostate biopsy were randomized to dutasteride or placebo. A 23% reduction in the rate of PC diagnosis was observed in the dutasteride arm after 4 years [57]. However, up to now neither finasteride nor dutasteride have been approved for chemoprevention for PC due to the lack of survival advantages. It has been suggested that 5ARI results in a higher proportion of tumors with higher Gleason grading [55]. However, no difference in mortality was shown between 5ARI users and nonusers within the same Gleason group in a large registry-based study [58]. This may be explained by a biopsy bias due to the shrinking prostates in treated men, or that 5ARI inhibits tumors with lower Gleason scores that would not have caused the death of the patients.

#### Higher levels of endogenous testosterone do not drive PC

Even if physiological levels of testosterone do not seem to be a risk factor for development of PC, another important question is whether it impacts the progression of a pre-existing PC, localized or metastasized. Several studies have addressed the risk of PC progression after radical prostatectomy (RP) in relation to baseline levels of testosterone. Almost unanimously, they suggest that higher levels of testosterone do not confer an increased risk of progression of localized PC [59–62]. The same appears to be valid for baseline levels of androgens before ADT, where also no associations or association with higher testosterone levels and poor prognosis have been reported [63, 64].

#### Saturation of the AR limits effect of testosterone

**Fig. 1:**
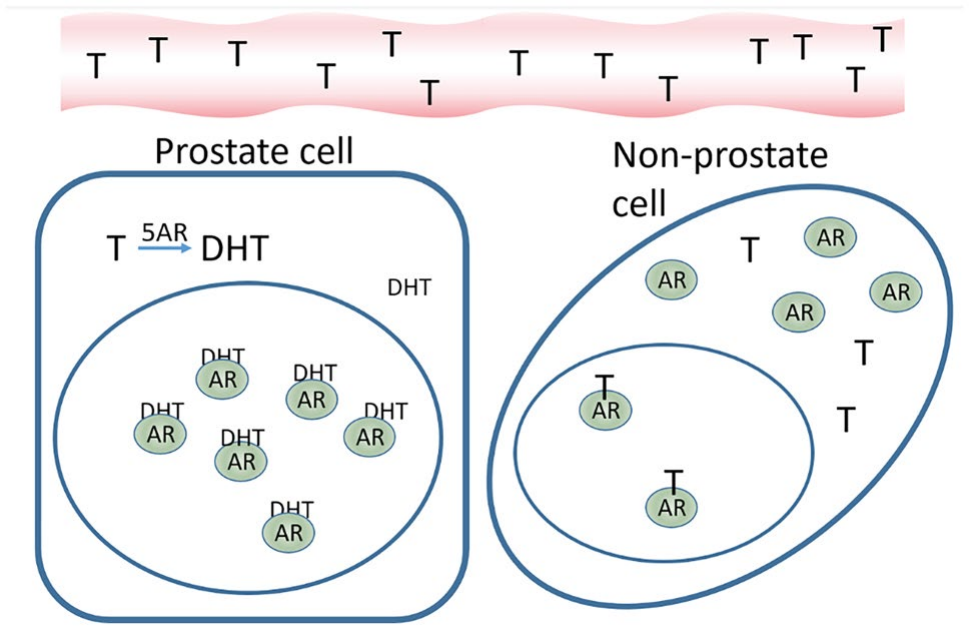
Simplified illustration of AR saturation in prostate cells. Translation of a certain testosterone (T) concentration in the circulation into saturated androgen receptor (AR) in prostate cells due to conversion into the more potent AR activator dihydrotestosterone (DHT) by 5-alpha reductase (5AR), compared to a relatively inefficient binding of testosterone to AR in non-prostate cells with lower 5AR activity. In the saturated prostate cell, all ARs are bound by ligand, while, at the same serum concentration of testosterone, a proportion of ARs in other organs is still free for activation

All these data collectively do not support a promoting role of testosterone on PC. One explanation for the apparent contradiction with the therapeutic effects of castration on PC, is the saturation model put forward by Morgentaler and Traish in 2009 [65], using extracted data from numerous studies as support. They relate to binding assays that show that prostate cells have a maximum of AR stimulation at 2–4 nM when all ARs are supposed to be bound by ligand, above which increase in testosterone results in negligible stimulation [66]. Supporting this, *in vitro* studies using PC cells also show a maximum stimulation of proliferation by testosterone approximately at 1 nM [67, 68]. The limited effect of concentrations of testosterone above this threshold was in line with studies in castrated rats supplemented with increasing doses of androgens, that showed rapid prostate regrowth in the lowest concentrations, above which a plateau was reached [69–71]. In eugonadal men, serum testosterone is 10-35nM, which is substantially higher than the saturation point for AR. In addition, the conversion of testosterone into DHT by 5AR in prostate cells may contribute to the rapid saturation of AR, since DHT has a higher affinity to AR compared to testosterone (Fig. 1). In line with this, several human studies show that increasing serum testosterone levels within the physiological range does not correlate with PSA secretion and prostate volume [72–76]. It has also been indicated that the intra-prostatic levels of androgens do not necessarily reflect changes in testosterone concentrations in the circulation [77, 78]. Rather constant testosterone and DHT concentrations in the prostate, despite increasing circulating levels in the physiological range, could also explain the lack of response to androgen modulations in the studies mentioned above. Together, this buffered prostatic environment and the saturation model for the AR provide robust evidence that modulating physiological testosterone levels does not significantly influence the androgenic stimulation of prostatic tissue.

#### Lower levels of endogenous testosterone as a risk factor for aggressive disease

Intriguingly, in some of the above-mentioned studies, lower testosterone was associated with more frequent aggressive high-risk disease [59, 79], and predicted poorer survival on ADT [63]. These associations do not fully fit into the saturation model, since even at these somewhat lower testosterone levels, the AR should be fully saturated and activated. Nevertheless, this connection was supported by the two studies mentioned above, where treatment with 5ARI was associated with fewer PC cases in total, but an increased proportion of high-risk tumors was seen [55, 57]. There are no experimental proofs for a dedifferentiation process of prostate cells initiated by these modest decreases in testosterone levels, but several studies have shown that castrate levels of testosterone (< 0.7 nM [80], below the saturation point) induce altered differentiation and increased metastatic potential of PC cells [81–83].

### Aging as a risk factor for prostate cancer

As mentioned above, age is the main risk factor for PC. Numerous studies have shown that the concentration of testosterone in men decreases with age, although the rate of decline differs somewhat between studies [84–86]. However, since the association with neither high nor low testosterone levels display a verified association with PC, there may be other factors than the age-related decrease in testosterone levels that affect the incidence of PC. Aging is associated with an increased risk for development of most cancer forms, since the total amount of mutations accumulate over the years [87], which has been demonstrated to be true also for prostate cancer cells [88]. The prostate is the only organ that continues to grow throughout the male life span. In line with this, in contrast to many organs [89, 90], old prostates maintain their progenitor capacity [91]. Actually, it has recently been demonstrated that the frequency of luminal cells with high progenitor activity are more frequent in prostates from older men [91, 92].

#### Age-related inflammation in PC development

Besides the increased mutational load, other age-related changes may be involved in the increased incidence of PC in elderly men. In animal studies, it has been shown that the numbers of T- and B-cells, as well as macrophages are increased in prostates from older mice compared to young mice, corresponding to a gene expression pattern indicative of a pro-inflammatory micro-environment [93]. In addition, old prostate cells of both epithelial and stromal origin display an inflammatory expression profile [94]. The association between PC and inflammation has been shown in numerous studies [95, 96]. Most convincing of a temporal association between chronic inflammation and the development of PC is a study conducted on the population included in the PCPT and SELECT studies [55, 97], where men in the untreated control arm who later developed PC (in the SELECT trial) displayed a higher degree of inflammation at baseline (i.e. at the end of PCPT trial) [98]. Interestingly, testosterone is believed to have an overall immunosuppressive role [99, 100], and despite the limited epidemiological evidence for its active role in PC development, testosterone may still influence immune cells and the inflammatory status of the prostate, irrespective of the saturation model working in prostate cells. This is supported by the observation that inflammation was more prominent among men treated with finasteride in the PCPT trial [98].

#### Aging of prostate microenvironment

The microenvironment is a crucial part of cancer biology. It constitutes the ecosystem in which the cancer cells develop and grow, and which some of them eventually leave to form metastases. In addition to the increased inflammation, structural changes appear in the prostatic microenvironment with age. These aging effects include changes in the collagen network as well as vascular remodeling [36, 93], features that might not be directly associated with PC initiation, but could affect both PC growth and differentiation [101, 102]. The prostate microenvironment can also work as a controlling and suppressing force, in the sense of maintaining normal organ function and thereby inhibiting excessive cancerous growth [103, 104]. In relation to aging, it has been shown that conditioned media from prostate stromal cells isolated from elderly men do not suppress the growth of prostate epithelial cells as efficiently, when compared to cells isolated from younger men [105].

#### The metabolic syndrome as risk factor for PC

As in the case with BPH, MetS is also associated with PC, as both are more frequent in older age, although the relation is more inexplicit [106]. MetS and its different aspects have been shown to be predictive for and play a role in the etiology of PC [107]. It is well known that T2DM, linked to both obesity and LOH [108], is associated with a lower number of incident PC [109, 110]. However, this may be a detection bias since diabetes is often associated with low PSA, in turn related to hypogonadism [111]. Nevertheless, an inverse relationship between T2DM and PC of different stage or grade has been demonstrated [112]. Besides T2DM, also obesity, especially visceral obesity, is an established aspect of MetS [24], and has been clearly linked to PC risk [113, 114]. In three large cohort studies a positive association between general adiposity and advanced PC was shown [115–117]. There are biological mechanisms that can explain the links between obesity and PC. Obesity is associated with increased levels of insulin-like growth factor-1, which promote PC and has been demonstrated to increased PC risk [118, 119]. On the cellular level, it was demonstrated that peritumoral adipocytes that secrete the chemokine CCL7 could facilitate migration and invasion of PC cells expressing CCR3, a receptor often upregulated in PC compared to normal prostate [120]. This can influence the spreading of PC cells outside the prostate and the establishment in the adipocyte rich bone marrow [121].

## Screening of prostate cancer

Population based screening for PC based on PSA testing is currently not recommended [122]. The outcome of two major randomized trials, including the European Randomised Study of Screening for Prostate Cancer (ERSPC) [123, 124] and Prostate, Lung, Colorectal and Ovary screening study (PLCO) [125] are not supportive for population screening using PSA, despite evidence of a reduction in PC-specific mortality. The inability of the PSA test to discriminate between clinically significant cancer and indolent cancers is a major limitation with PSA based screening. The harms of unnecessary prostate biopsies resulting in overdiagnosis and over treatment of indolent cancers outweigh any benefits of potential mortality reduction. Today, most guidelines recommend informing men over 50 years of age about pros and cons with PSA testing, so they can make informed decisions based on their individual situation, while risk-adapted strategies often are applied for men confirmed to be of higher risk due to genetic profiles or family history. Recent developments of magnetic resonance imaging (MRI)-fusion technologies for prostate imaging, which can classify men with elevated PSA into different risk categories, leading to biopsies only on men with risk for clinically significant cancer, appears as a promising concept for future screening strategies. This concept is presently tested in prospective clinical trials [126, 127] and recently it was shown that a PSA-based blood test (Stockholm3) combined with MRI- targeted biopsy decreased over detection while maintaining the ability to detect clinically significant PC in a prospective randomized screening trial [128].

### PC screening in men with hypogonadism

Even if screening of the general population is not widely implemented, PSA is commonly clinically used as a marker in the diagnosis of PC [129]. Although the exact relationship between testosterone and PC is unclear, the production of PSA is under testosterone influence and untreated patients with hypogonadism have generally lower PSA compared to eugonadal men [130]. Furthermore, a low PSA has been suggested as a marker for hypogonadism [131, 132]. Consequently, when using PSA as a screening tool for PC in untreated hypogonadal men, the PSA cut off value for further investigations should probably be held at a lower level since the number of detected PC was high in a group hypogonadal men with PSA below 4 ng/ml [133]. In hypogonadal men on TRT, it was shown that PSA levels after an initial increase remain stable after normalization of testosterone for 5 years, and, in addition, PC could be effectively diagnosed and treated in men taking TRT using the standard cut-off levels [134]. Due to continuing uncertainties regarding risks/benefits of PC screening, men with LOH on TRT should be offered the option of prostate safety monitoring before and during treatment [135] including digital rectal examination (DRE) and PSA, using the same cut-off values as eugonadal men for extended investigations. Taken together, men on TRT can be screened with PSA in a similar way as the general male population.

## Prostate health in men with TRT

### TRT and risk of developing BPH

As described above, it appears that androgens in some ways are involved in the development of BPH. It is also known that BPH is strongly related to age, and during aging, serum androgens are decreasing indicating that also other factors are involved in BPH development [136]. Furthermore, in a study of more than 4000 men with BPH, no association between PSA, prostate size and testosterone levels were found [137]. In a systematic review of the literature, it was clearly demonstrated that in men with LOH, TRT did not have any influence on prostate size [138]. In a randomized trial, TRT was also associated with improvement of lower urinary tract symptoms, which are complaints strongly associated with BPH in aging males [139]. Although patients with severe LUTS were excluded from most studies, there appears not to be any increased risk for BPH/LUTS in patients on TRT.

### TRT and risk of developing prostate cancer

Restoring testosterone levels in med with LOH has long been associated with a fear of inducing PC. However, it has been demonstrated that the incidence of PC among men on TRT was not higher than that in the general population [134]. Although there is no level one evidence, there are several large observational studies supporting that hypogonadal men on TRT do not have an increased risk for PC diagnosis. A study including 750 men on TRT found no increased risk of PC [140], which was supported by a larger study including almost 13,000 men also demonstrating no association between TRT [141]. In addition, a lower proportion of aggressive PC cases in the TRT group has been shown [142]. Taken together, all these studies point to, in line with the saturation model, that restoring testosterone levels by TRT in hypogonadal men does not increase the risk of PC. However, while waiting for more conclusive evidence, it is reasonable to offer PSA monitoring on a regular basis during TRT, as well as to check PSA before initiating TRT.

### TRT in men diagnosed with PC

There is growing evidence of health benefits of TRT for men with LOH. However, concurrent or historical, PC has conventionally been considered as an absolute contraindication for TRT for these men. In clinical practice, this standpoint is gradually starting to change since normalization of serum testosterone levels does not appear to drive PC progression in men with LOH treated with testosterone.

#### TRT in men with low-risk PC

Active surveillance (AS) is the choice of treatment strategy for patients with low-risk prostate cancer [143]. To date, there are no randomized controlled trials that investigated the risk of TRT in men on AS. In a retrospective cohort study of 82 hypogonadal men with PC on AS and on TRT followed for up to 41 months, despite a small increase in PSA, no patients progressed to a higher Gleason grade on subsequent biopsies [144]. A retrospective observational study including 13 hypogonadal men with low-risk PC on AS and TRT for a 2.5-year median treatment duration, showed neither PSA nor prostate volume changes, and no PC progression was observed [145]. In another study, it was shown that the biopsy progression rates in testosterone-deficient men on AS was similar in 28 men on TRT compared to 96 untreated men [146].

To summarize, in hypogonadal men on AS for low stage/grade PC, the current body of evidence supporting TRT is restricted to a few small, retrospective observational studies. Taken together, these studies are not conclusive regarding the safe use of TRT in hypogonadal men on AS, as some studies reported high progression rates for patients on TRT during AS [147]. Prospective randomized clinical trials in this field are needed.

#### TRT in men treated for PC

RP and radiotherapy are effective and common approaches for treatment of localized PC [143]. The question is whether TRT is safe for men with LOH previously treated for localized PC.

In a small case series on a group of men treated with RP and without biochemical recurrence [148], it was reported that after TRT, the PSA remained undetectable. In another retrospective study of men treated for LOH after RP, no biochemical recurrences were noted during the follow up of 7–18 months [149]. In a mixed cohort of patients with both high and low risk PC treated with RP, including patients on TRT because of testosterone deficiency followed for 36 months, a PSA increase was observed in the TRT group, but with more biopsy verified PC-recurrences in the group without TRT [150]. Although the lack of sufficiently large and lengthy prospective trials is obvious, these two observational studies suggest that there is no apparent increase in PC recurrence in men treated with TRT after RP.

For radiotherapy, such as external beam radiation or brachytherapy, data regarding TRT is sparse. In an early study from 2007, it was concluded that no biochemical recurrence occurred in patients treated with brachytherapy and subsequently with testosterone due to LOH [151]. In a larger retrospective study, 98 men with PC and treated with radiotherapy and subsequently given testosterone for LOH, a clinically insignificant increase in mean PSA was observed [152]. Also, for radiotherapy, the sparse data available does not show that the use of TRT increase the risk of worsen PC severity. In a recent systematic review of available literature [147], eight out of nine included studies showed that TRT-treated patients with PC treated by radiation therapy did not have an increased risk for disease progression. However, the overall quality of available evidence is poor and prospective controlled studies are lacking.

In the same systematic review, seven out of nine included studies indicated that TRT appears to be harmful for patients with advanced PC, although the quality of the included studies was poor [147]. Thus, despite the non-conclusive nature of the evidence, TRT should not be recommended for this patient group.

In summary, despite the potentially harmful role of TRT in advanced PC, the risks with TRT for patients with low-risk and treated localized PC appear to be small. However, since this cannot be concluded based on current evidence, whether TRT should be initiated in individual PC patients needs to be carefully considered, while careful monitoring of PC patients on TRT is strongly recommended for safety reasons (Table 1).

**Table 1:** Overview of TRT in relation to prostate health issues

**Prostate health situation**	**Interpretation of current data**	**Comments**
General PSA screening programs	Patients on TRT can be included	Lower PSA cut-off may be considered
BPH and TRT	No concerns	Could even be beneficial
PC development and TRT	Data indicate no increased risk	Randomized controlled trials are lacking
Low-risk PC and TRT	No observed risk for progression	Evidence is sparse
Localized PC and TRT		
* after radical prostatectomy*	No observed increased risk for relapse	Observational studies only
* after radiotherapy*	No observed increased risk for relapse	Evidence is sparse
Advanced PC and TRT	TRT potentially harmful	Not recommended

*TRT* testosterone replacement therapy, *PSA* prostate specific antigen, *BPH* benign prostate hyperplasia, *PC* prostate cancer

## Potential role of selective androgen receptor modulators (SARMs) in prostate health

As evidenced by the effect of modulating its levels, testosterone is a powerful mediator of both anabolic and androgenic effects. When aiming to target only parts of its repertoire, such as inhibiting PC growth without inducing the side-effects of systemic castration or restoring systemic anabolic effects without possible negative impact on the prostate, testosterone itself may be a too blunt instrument. Selective androgen receptor modulators (SARMs), steroidal or nonsteroidal molecules with agonistic or antagonistic action, are being developed with the hope to enable tissue-specific effects on AR activity. Besides optimized AR modulating functions, these substances may also display improved bioavailability and pharmacology profiles.

### Therapeutic potential of SARMs in PC

The AR activates different genes in different cell types, however, a specific mechanism for this has not been clearly defined. Binding of different ligands, different co-factor interactions [153, 154], as well as different concentration of the ligand may impact the transcription profile [155–157]. Despite considerable efforts, no SARMs can so far fully discriminate between desirable and non-desirable effects in certain situations (as reviewed in [158]). Considering the small effect on the prostate within physiological levels of testosterone, the need of SARMs may be limited in the treatment of men with LOH.

Non-steroidal antiandrogens (e.g., bicalutamide) are commonly used in treatment of PC. There are also new antiandrogens, second generation androgen receptor inhibitors (enzalutamide, apalutamide), which are used in treatment of advanced PC. Anti-androgens are not used for treatment of BPH. However, when aiming to efficiently block AR signaling in metastatic PC and castration resistant PC, SARMs that do not confer systemic side-effects such as metabolic syndrome and osteoporosis would be important therapeutic tools.

## Summary

There is an increased usage of TRT in the aging male population mostly due to LOH, a condition associated with important health deteriorations. Since prostate is a target organ for androgens and the anti-androgenic strategies that are used to treat and palliate BPH and PC, the possible influence of TRT on prostate health becomes apparent.

The present review summarizes existing data on the associations between endogenous hormone concentrations and prostate growth and concludes that circulating concentrations of androgens do not appear to be associated with the risk of development of BPH. On the contrary, TRT appears to be associated with improvement of BPH associated symptoms. It becomes obvious that other factors than androgens are crucial for development of clinical BPH, such as the metabolic syndrome and its components, inflammation, and cellular senescence.

Analysis of existing data on the associations between endogenous hormone concentrations and PC, indicates that circulating androgens do not appear to be associated with the risk of PC initiation or progression. The explanation for this apparent controversy with the efficacy of anti-androgen strategies of PC, could relate to the prostate AR saturation model, which can explain why physiological levels of testosterone do not affect PC cells. PSA levels are in general lower in men with LOH and may mask a cancer. Therefore, monitoring of PSA before and during TRT is recommended. Patients on TRT should be treated as eugonadal men regarding prostate check-up, meaning that MRI and biopsies should be performed with the same indications (at the same PSA values). TRT of hypogonadal men does not in general increase the risk for PC. Men with low-risk PC or patients cured from PC, and with hypogonadism, could probably safely be treated with TRT, while in patients with advanced prostate cancer a more restricted attitude to TRT is reasonable.
